# Editorial

**DOI:** 10.4102/curationis.v42i1.2133

**Published:** 2019-12-12

**Authors:** Mokgadi Matlakala

**Affiliations:** 1 Department of Health Studies, College of Human Sciences, University of South Africa, Pretoria, South Africa

In this issue, Vol 42, No 1 (2019), the journal presents articles that address stimulating contemporary issues in nursing. There are articles with specific focus on primary healthcare, clinical learning environment, and nursing education and training. The focus on primary health resonates with primary health re-engineering in South Africa. The evidence from the articles on this theme provides support for the need to strengthen the district health system. The primary healthcare focus has the potential of enhancing clinical practitioners’ interest in research-based practice. The evidence for clinical learning environment provides support for quality improvement in nursing practice. With regard to nursing education and training, the issue draws attention to debates on the impact of the policy related to the new qualifications for the nursing profession. The articles outline important aspects of the position of nursing education in South Africa, one of which relates to nursing colleges having to be registered as higher education institutions in order to have their offerings on a higher qualification band. This is in line with the transformation of nursing education in South Africa. It is believed that the evidence presented in these articles will encourage further debates on the status and process of transformation in nursing education and training in South Africa and internationally. The combination of articles indicates the wealth of scientific knowledge that is produced in nursing for the profession and its practice. The journal strives to continue to produce scientific knowledge that is thought-provoking.

